# Dominance Effects and Functional Enrichments Improve Prediction of Agronomic Traits in Hybrid Maize

**DOI:** 10.1534/genetics.120.303025

**Published:** 2020-03-09

**Authors:** Guillaume P. Ramstein, Sara J. Larsson, Jason P. Cook, Jode W. Edwards, Elhan S. Ersoz, Sherry Flint-Garcia, Candice A. Gardner, James B. Holland, Aaron J. Lorenz, Michael D. McMullen, Mark J. Millard, Torbert R. Rocheford, Mitchell R. Tuinstra, Peter J. Bradbury, Edward S. Buckler, M. Cinta Romay

**Affiliations:** * Institute for Genomic Diversity, Cornell University, Ithaca, New York 14853; † Section of Plant Breeding and Genetics, Cornell University, Ithaca, New York 14853; ‡ Division of Plant Science, University of Missouri, Columbia, Missouri 56211; § U.S. Department of Agriculture–Agricultural Research Service, Ames, Iowa 50011; ** Syngenta Seeds, Stanton, Minnesota 55018; †† U.S. Department of Agriculture–Agricultural Research Service, University of Missouri, Columbia, Missouri 56211; ‡‡ U.S. Department of Agriculture–Agricultural Research Service, Department of Crop and Soil Sciences, North Carolina State University, Raleigh, North Carolina 27695; §§ Department of Agronomy and Horticulture, University of Nebraska, Lincoln, Nebraska 68588; *** Department of Agronomy, Purdue University, West Lafayette, Indiana 47907; ††† U.S. Department of Agriculture–Agricultural Research Service, Ithaca, New York 14853

**Keywords:** dominance, genomic features, functional enrichment, genomic prediction, hybrid maize

## Abstract

Single-cross hybrids have been critical to the improvement of maize (*Zea mays* L.), but the characterization of their genetic architectures remains challenging. Previous studies of hybrid maize have shown the contribution of within-locus complementation effects (dominance) and their differential importance across functional classes of loci. However, they have generally considered panels of limited genetic diversity, and have shown little benefit from genomic prediction based on dominance or functional enrichments. This study investigates the relevance of dominance and functional classes of variants in genomic models for agronomic traits in diverse populations of hybrid maize. We based our analyses on a diverse panel of inbred lines crossed with two testers representative of the major heterotic groups in the U.S. (1106 hybrids), as well as a collection of 24 biparental populations crossed with a single tester (1640 hybrids). We investigated three agronomic traits: days to silking (DTS), plant height (PH), and grain yield (GY). Our results point to the presence of dominance for all traits, but also among-locus complementation (epistasis) for DTS and genotype-by-environment interactions for GY. Consistently, dominance improved genomic prediction for PH only. In addition, we assessed enrichment of genetic effects in classes defined by genic regions (gene annotation), structural features (recombination rate and chromatin openness), and evolutionary features (minor allele frequency and evolutionary constraint). We found support for enrichment in genic regions and subsequent improvement of genomic prediction for all traits. Our results suggest that dominance and gene annotations improve genomic prediction across diverse populations in hybrid maize.

SINCE the development of the first maize hybrids by Shull (1908) and their widespread adoption starting in the 1930s, hybrids have been central to the improvement of maize in the U.S. Prevailing hypotheses about their advantages have focused on complementation of parental genomes (Crow 1998). The basis for such complementation consists of nonadditive genetic effects, particularly dominance (within-locus complementation, *i.e.*, interaction between alleles within single genetic loci) and epistasis (among-locus complementation, *i.e.*, interactions involving multiple genetic loci). Dominance has been proposed as a major driver of genomic complementation in maize hybrids (Crow 1998; Lamkey and Edwards 1999). Epistasis also provides a plausible explanation for genomic complementation, but studies assessing its contribution to hybrid advantage have suffered from a lack of statistical power (Reif *et al.* 2005) and have reported conflicting results (*e.g.*, Mihaljevic *et al.* 2005; Ma *et al.* 2007).

Genetic studies in maize have investigated dominance gene action by focusing either on directional dominance (consistent dominance effects across loci), effects of significant quantitative trait loci (QTL), or genome-wide polygenic effects. Studies on testcrosses or diallel mating designs seem to support the presence of directional dominance, particularly for grain yield (GY) (Hinze and Lamkey 2003; Reif *et al.* 2003). Furthermore, studies on populations derived from backcrosses between recombinant inbred lines (RILs) and their parents have generally identified several QTL with significant dominance effects for traits such as flowering time, plant height (PH), and GY (*e.g.*, Frascaroli *et al.* 2007; Larièpe *et al.* 2012). However, genomic prediction analyses often have not shown the contribution of dominance effects to genotypic variability, because they focused on populations of hybrids obtained from crosses between heterotic groups: Flint and Dent (*e.g.*, Technow *et al.* 2014), or Stiff Stalk (SS) and non-Stiff Stalk (NSS) (*e.g.*, Kadam *et al.* 2016). Assessing the relevance of dominance effects in genomic prediction models would require more diverse panels in which complementation effects are more variable, due to differential degrees of complementarity within and across heterotic groups (Reif *et al.* 2005; Gerke *et al.* 2015).

The studies above have examined the relative importance of additive and dominance effects across the genome, but have not attempted to describe the properties of loci most enriched for causal variants: gene proximity, structural features, and/or evolutionary features. Gene proximity has been linked to causal variants in maize through enrichment for QTL effects in genic regions (Wallace *et al.* 2014), consistent with a large portion of variability of gene expression being attributed to *cis* polymorphisms in maize (Schadt *et al.* 2003). Structural features such as chromatin openness and high recombination rate were also associated with enrichment for QTL effects in maize inbred lines (Rodgers-Melnick *et al.* 2016), but studies on hybrids have also shown that dominance effects could locate around centromeres, where recombination is low (Larièpe *et al.* 2012; Thiemann *et al.* 2014, Martinez *et al.* 2016). Evolutionary features reflecting low allelic diversity within (allele frequency or nucleotide diversity) and across species (evolutionary constraint) have been associated with stronger QTL effects in hybrid maize (Mezmouk and Ross-Ibarra 2014; Yang *et al.* 2017). Importantly, structural and evolutionary features have also been associated with gene proximity (Beissinger *et al.* 2016; Rodgers-Melnick *et al.* 2016). Therefore, there is ambiguity about the relevance of evolutionary and structural features to capture variability at agronomic traits independently from gene proximity.

In this study, we characterized the genetic basis of three agronomic traits—days to silking (DTS), PH, and GY—in panels representative of the diversity in North-American hybrid maize. We analyzed two hybrid panels. One was derived from crosses between a diverse sample of maize inbred lines and either of two testers, B47 and PHZ51, belonging respectively to the SS and NSS heterotic groups. The other was derived from crosses between the U.S. Nested Association Mapping panel and PHZ51. Our study investigated the determinants of genotypic variability in hybrid maize, based on gene action (additive and/or dominance effects) or functional enrichments (by gene proximity and structural or evolutionary features) (Figure 1).

**Figure 1:**
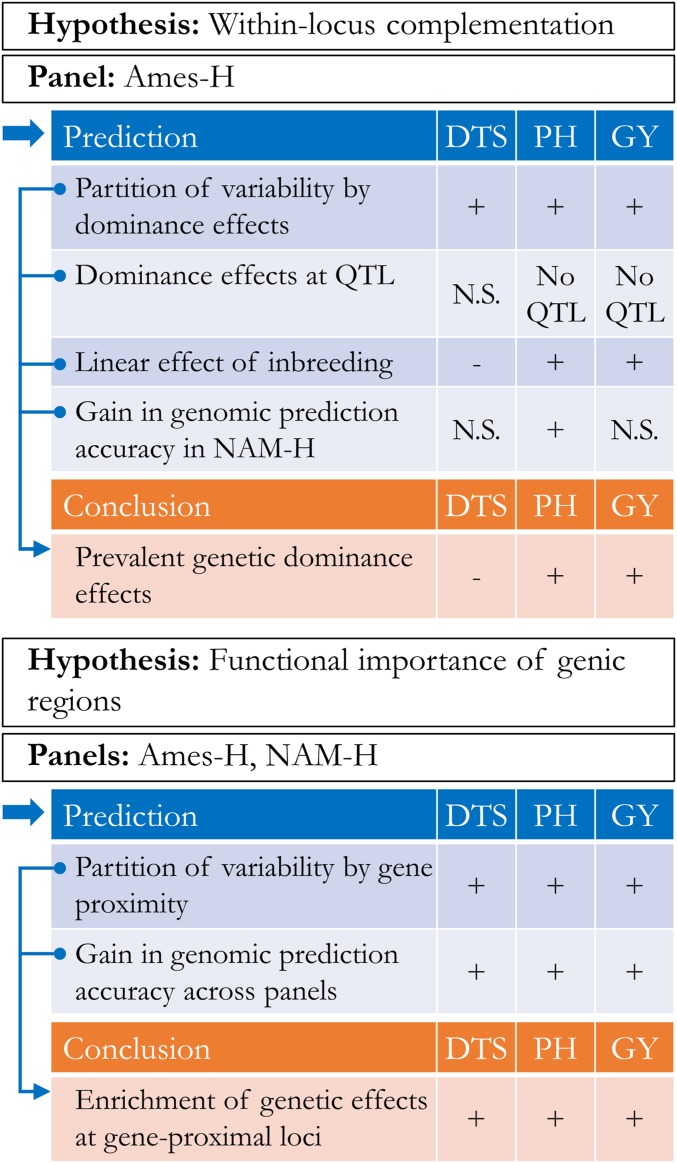
Graphical summary of the study. We tested hypotheses regarding the statistical importance of two attributes of genomic variability in hybrid maize: (1) dominance effects and (2) enrichment of genetic effects in genic regions. Under each hypothesis, evidence from analyses is characterized as consistent (+) or inconsistent (−). Nonconclusive evidence is either due to absence of QTL (no QTL) or lack of significance (N.S.). Ames-H, hybrid panel from Ames, North Central Regional Plant Introduction Station association panel; NAM-H, hybrid panel from NAM, U.S. Nested Association Mapping panel. DTS, days to silking; PH, plant height; GY, grain yield adjusted for DTS.

## Materials and Methods

### Phenotypic data

#### Phenotypic measurements:

In this study, two panels of maize lines were evaluated for hybrid performance: the North Central Regional Plant Introduction Station association panel (hereafter, Ames) and the U.S. Nested Association Mapping panel (hereafter, NAM). The Ames panel is a subset of temperate inbred lines from the diversity panel described by Romay *et al.* (2013); the NAM panel is a subset of 24 RIL populations, all having one parent in common, B73, as described by McMullen *et al.* (2009).

The hybrid Ames panel (Ames-H) was derived from a subset of 875 inbred lines, which were selected to reduce differences in flowering time while favoring genetic diversity based on pedigree. Two inbred lines were selected as testers: one NSS inbred (PHZ51) and one SS inbred (B47, also known as PHB47). Inbreds were assigned to one or two testers based on known heterotic group: SS inbreds were crossed with PHZ51, while NSS inbreds were crossed with B47; inbreds with unknown heterotic group, as well as inbreds belonging to the Goodman association panel (Flint-Garcia *et al.* 2005), were crossed with both testers, for a total of 1111 hybrids. The hybrid NAM panel (NAM-H) was developed as described by Larsson *et al.* (2017 *preprint*). Briefly, a subset of up to 80 RILs from each of the NAM families was selected to reduce differences in flowering time across families: the later RILs from the earliest families and the earlier RILs from the latest families, for a total of 1799 RILs. All NAM RILs were crossed with the same tester, PHZ51.

In Ames-H, evaluations were performed in 2011 and 2012, at six locations across the U.S.—Ames (IA), West Lafayette (IN), Kingston (NC), Lincoln (NE), Aurora (NY), and Columbia (MO)—for a total of nine environments: 11IA, 11IN, 11NC, 11NE, 11NY, 11MO, 12NE, 12NC, and 12MO. In NAM-H, hybrids were evaluated at five locations—Ames (IA), West Lafayette (IN), Kingston (NC), Aurora (NY), and Columbia (MO)—during 2010 and 2011 for a total of eight environments: 10IA, 10IN, 10NC, 10MO, 11IA, 11IN, 11NC, and 11NY. The following traits were measured in each hybrid panel: DTS (number of days from planting until 50% of the plants had silks), PH (centimeters from soil to flag leaf), and GY (tons per hectare adjusted to 15.5% moisture). In 11NY, only PH and DTS were measured (Table 1). Both Ames-H and NAM-H panels were planted in two-row plots (40–80 plants per plot; 50,000–75,000 plants per hectare), except for 11NY, where 12 plants were planted per plot.

**Table 1:** Phenotypic information by panel and trait

Panel	Trait	Environments	Mean	$\sigma_{g}$	H^2^	$r_{g}^{2}$
Ames-H	DTS	11IA 11IN 11NC 11NE 11NY 11MO	66.4	2.1	0.78	0.95
		12NC 12NE 12MO				
	PH	11IA 11IN 11NC 11NE 11NY 11MO	219	13	0.69	0.92
		12NC 12NE 12MO				
	GY	11IA 11NC 11NE 11MO	6.13*^a^*	0.66	0.29	0.62
		12NC 12NE 12MO				
NAM-H	DTS	10IA 10IN 10MO	70.6	1.5	0.55	0.81
		11IA 11IN 11NC 11NY				
	PH	10IA 10IN 10NC 10MO	247	10	0.30	0.69
		11IA 11IN 11NC 11NY				
	GY	10IA 10IN 10MO	6.94^a^	0.44	0.16	0.35
		11IA 11NC				

Environments refer to year (2010, 2011, and 2012) and locations [Kingston (NC), Ames (IA), West Lafayette (IN), Lincon (NE), Columbia (MO), and Aurora (NY)]. Mean: average phenotypic value; $\sigma_{g}$, genotypic SD; H^2^, broad-sense heritability on a plot basis; $r_{g}^{2}$, average entry-mean reliability. Ames-H, hybrid panel from Ames, North Central Regional Plant Introduction Station association panel; NAM-H, hybrid panel from NAM, U.S. Nested Association Mapping panel. DTS, days to silking; PH, plant height; GY, grain yield adjusted for DTS.

a Means shown for GY are adjusted by mean DTS.

Phenotypic evaluations were conducted under augmented block designs, where blocking was used to reduce competition for light due to heterogeneous height and/or phenology (only checks were replicated within environments). In Ames-H, blocks were combinations of tester (PHZ51 or B47), maturity (early or late), and expected PH (short, medium, or tall); checks were B73 × PHZ51 (up to six replicates per block) as well as PHZ51 × B47, B47 × PHZ51, and a maturity commercial check (each replicated once per block). In NAM-H, blocks were NAM families; checks were B73 × PHZ51 (≤ 15 replicates per block) and the non-B73 parent crossed with PHZ51 (≤ 7 replicates per block) (Larsson *et al.* 2017 *preprint*).

#### Genotype means and heritability:

For each combination of panel (Ames-H or NAM-H) and trait (DTS, PH, or GY), genotype means of hybrids were estimated by the following linear mixed model, fitted by ASREML-R v3.0 (Butler *et al.* 2009):

$$y_{ijkl}=g_{i}+{\left(Env.\right)}_{jk}+{\left(Field\right)}_{jkl}+s_{ijkl}+e_{ijkl}$$

where $g_{i}$ was the mean of genotype *i* (fixed), ${\left(Env.\right)}_{jk}$ was the effect of location *j* and year *k* [random, independent, and identically normally distributed (i.i.d.)], ${\left(Field\right)}_{jkl}$ was the effect of field *l* within environment *jk* (random, i.i.d.), $e_{ijkl}$ was the error (random, i.i.d.), and $s_{ijkl}$ was a spatial effect within environment–field combinations (random, normally distributed under first-order autoregressive covariance structures by row and column). Since genotypes were not replicated within environments, genotype-by-environment interactions were pooled with errors. For PH in both panels, spatial effects were not included in the model because the fitting algorithm could not converge to a solution. For GY in both panels, DTS measurements (fixed) were included in the model to account for phenological differences among lines. In addition to estimating genotype effects (*g_i_*’s) as fixed, models with genotype effects as random were also fitted to estimate genotypic variance $\left(\sigma_{g}^{2}\right)$ and error variance $\left(\sigma_{e}^{2}\right)$. Broad-sense heritability on a plot basis was then calculated as $H^{2}=\frac{\sigma_{g}^{2}}{\sigma_{g}^{2}+\sigma_{e}^{2}}$. Finally, the average entry-mean reliability was estimated as $r_{g}^{2}=1-\frac{1}{n}\overset{n}{\underset{i=1}{\sum }}\frac{\text{Var}\left(g_{i}-{\hat{g}}_{i}\right)}{\sigma_{g}^{2}}$, where *n* is the number of hybrids evaluated in either panel and $\text{Var}\left(g_{i}-{\hat{g}}_{i}\right)$ is the prediction error variance of the random genotype effect for hybrid *i* (Searle *et al.* 2009).

### Genotypic data

#### Marker data:

Whole-genome sequencing (WGS) SNPs were previously called in the Hapmap 3.2.1 reference panel (Bukowski *et al.* 2018), under version 4 of the B73 reference genome. In this study, marker scores (allele counts) at WGS SNPs in the Ames and NAM inbred panels were imputed from genotyping-by-sequencing (GBS) SNPs (Romay *et al.* 2013; Rodgers-Melnick *et al.* 2015). Then, marker scores at WGS SNPs in Ames-H and NAM-H hybrids were inferred based on the marker scores at their respective parents in the Ames and NAM inbred panels.

In the Ames and NAM inbred panels, GBS SNPs were called with the software TASSEL v5.0 (Bradbury *et al.* 2007) using the GBS production pipeline and the ZeaGBSv2.7 TagsOnPhysicalMap files (Glaubitz *et al.* 2014). In the Hapmap 3.2.1 reference panel, WGS SNPs were processed as follows: 25,555,019 SNPs were selected (two alleles per SNP, call rate ≥ 50%, and minor allele count ≥ 3), heterozygous marker scores were set to missing (since these were presumably due to errors or collapsed paralogous loci), and missing marker scores were imputed. Marker scores at WGS SNPs in the Ames and NAM inbred panels were then imputed from GBS SNPs (separately by panel), using the Hapmap 3.2.1 panel WGS SNPs as reference. Imputations of marker scores at WGS SNPs were performed by BEAGLE v5 (Browning *et al.* 2018), with 10 burn-in iterations, 15 sampling iterations, and effective population size set to 1000.

After imputation, *m* = 12,659,487 WGS SNPs were selected for estimated squared correlation between imputed and actual marker scores (≥ 0.8; Browning and Browning 2009) and minor allele frequency (≥ 0.01), in each combination of inbred panel and tester (*e.g.*, set of Ames inbreds crossed to PHZ51), to avoid SNPs private to any of these sets. Marker scores at selected WGS SNPs were then inferred for each hybrid by CreateHybridGenotypesPlugin in TASSEL v5.0. The marker data at selected WGS SNPs in Ames-H (*n* = 1106) and NAM-H (*n* = 1640) consisted of the matrix **X** of minor allele counts, where minor alleles were defined by frequencies in the Hapmap 3.2.1 panel, and the matrix **Z** of heterozygosity, which coded homozygotes as 0 and heterozygotes as 1.

#### Population principal components:

Principal component (PC) analysis (PCA) was performed using the R package irlba v2.3.3 (Baglama and Reichel 2005) in the Goodman panel representing the genetic diversity among elite maize inbred lines (Flint-Garcia *et al.* 2005). The first three PCs were computed based on allele counts at selected WGS SNPs (4.7%, 3.2%, and 2.1% of genomic variability explained in the Goodman panel, respectively). The matrix **P** of coordinates at the first three PCs in Ames-H and NAM-H was obtained by: (i) adjusting allele counts by their observed mean in the Goodman panel and (ii) mapping adjusted allele counts to PCs by SNP loadings, *i.e.*, P=(X−M)V, where X−M is the matrix of adjusted allele counts in hybrid panels and **V** is the m×3 matrix of right-singular vectors from the PCA.

### Functional features

#### Gene annotation (proximity to genes):

Gene positions were available from v4 gene annotations, release 40 (ftp://ftp.ensemblgenomes.org/pub/plants/release-40/gff3/zea_mays/Zea_mays.AGPv4.40.gff3.gz). Gene proximity bins (either “proximal” or “distal”) indicated whether any given SNP was within 1 kb of an annotated gene (< 1 kb away from start or end positions).

#### Structural features (recombination rate and chromatin openness):

Published recombination maps identified genomic segments originating from either parent within the progeny of each NAM family (Rodgers-Melnick *et al.* 2015). These maps were uplifted to version 4 of the reference genome using CrossMap v0.2.5 (Zhao *et al.* 2014). Then, the recombination fractions were fitted on genomic positions by a thin-plate regression spline model, using the R package mgcv v1.8-27 (Wood 2003). Based on this model, recombination rates **c** were inferred by finite differentiation of fitted recombination fractions: $c=f\left(s+\frac{1}{2}\right)-f\left(s-\frac{1}{2}\right)$, where s is the vector of genomic positions of all WGS SNPs and *f* is the function inferred by the spline model. We defined recombination bins as follows: *c_j_* ≤ 0.45 cM/Mb, 0.45 cM/Mb < *c_j_* ≤ 1.65 cM/Mb, and 1.65 cM/Mb < *c_j_*, where 0.45 cM/Mb and 1.65 cM/Mb are the first two tertiles of estimated recombination rates among all WGS SNPs.

Chromatin accessibility was measured by micrococcal nuclease hypersensitivity (MNase HS) in juvenile root and shoot tissues in B73 (Rodgers-Melnick *et al.* 2016). Here, MNase HS peaks were mapped to their coordinates in version 4 of the reference genome. A given SNP was considered to lie in a euchromatic (open) region if an MNase HS peak was detected, in either root or shoot tissues. We defined MNase HS bins as “dense” or “open” for the absence or presence of MNase HS peaks, respectively.

#### Evolutionary features (minor allele frequency and evolutionary constraint):

Minor allele frequency (MAF) at SNPs was determined in the Hapmap 3.2.1 panel. Similarly to Evans *et al.* (2018), we defined MAF bins as follows: MAF ≤ 0.01, 0.01 < MAF ≤ 0.05, and 0.05 < MAF (SNPs were not binned at MAF ≤ 0.0025 due to only 7202 of them falling into this class).

Evolutionary constraint at SNPs was quantified by genomic evolutionary rate profiling (GERP) scores (Davydov *et al.* 2010), derived from a whole-genome alignment of 13 plant species (Rodgers-Melnick *et al.* 2015; Yang *et al.* 2017), in version 4 of the reference genome. We defined GERP score bins by GERP ≤ 0 and GERP > 0.

### Genomic models

#### Polygenic additive effects (genomic best linear unbiased prediction):

Genome-wide additive effects were estimated under a genomic best linear unbiased prediction (GBLUP) model (VanRaden 2008), as follows:

$$g=Q\delta +u+\varepsilon ;u∼N\left(0,G\sigma_{u}^{2}\right);\varepsilon ∼N\left(0,I\sigma_{\varepsilon }^{2}\right);G=XX^{’}/m$$

where **g** was the vector of genotype means; $Q=\left[\begin{array}{cc} 1 & P \end{array}\right]$ was the matrix consisting of a vector of ones and the three PCs as described above; δ were fixed effects associated to **Q**; **u** and ε consisted of polygenic additive effects and random errors, respectively; and **X** was the (noncentered) matrix of minor allele counts at m=12,659,487 WGS SNPs as described above. The GBLUP model was fitted in Ames-H or NAM-H by restricted maximum likelihood (REML) using the R package regress v1.3-15 (Clifford and McCullagh 2005).

#### Polygenic additive and dominance effects (dominance GBLUP):

To account for dominance, the GBLUP model was extended to the dominance GBLUP (DGBLUP) model, as follows:

$$g=Q\delta +u+w+\varepsilon ;u∼N\left(0,G\sigma_{u}^{2}\right);w∼N\left(0,D\sigma_{w}^{2}\right);\varepsilon ∼N\left(0,I\sigma_{\varepsilon }^{2}\right);G=XX^{’}/m;D=ZZ^{’}/m$$ (1)

where **w** consisted of polygenic dominance effects and **Z** was the (noncentered) matrix of heterozygosity at m=12,659,487 WGS SNPs as described above. Notably, in NAM-H, additive effects were completely confounded with dominance effects, because only one inbred tester was used in this panel (and only two genotype classes could exist at any given marker). Therefore, model (1) was fitted only in Ames-H by REML using the R package regress v1.3-15 (Clifford and McCullagh 2005).

#### Directional effects:

Under a model of directional dominance without linkage or epistasis, inbreeding depression is characterized by a linear negative relationship between the inbreeding coefficient and fitness (Falconer and Mackay 1996). In the presence of directional epistatic effects, the relationship between the inbreeding coefficient and fitness is expected to be nonlinear (Crow and Kimura 1970). To capture such nonlinearity, especially dominance × dominance epistasis, the quadratic effect of the inbreeding coefficient was fitted along with its linear effect in genomic models. We followed Endelman and Jannink (2012) to estimate genomic inbreeding coefficients with respect to a base population, here represented by the Goodman panel. For each hybrid *i*, the coefficient of genomic inbreeding was calculated as $F_{i}=\frac{\sum_{j}{\left(x_{ij}-2\pi_{j}\right)}^{2}}{\sum_{j}2\pi_{j}\left(1-\pi_{j}\right)}-1$, where $\pi_{j}$ was the allele frequency in the Goodman panel.

Directional effects of inbreeding were analyzed as fixed effects under an extension of the DGBLUP model (1). The following model was fitted:

$$g=Q\delta +R\tau +u+w+\varepsilon ;u∼N\left(0,G\sigma_{u}^{2}\right);w∼N\left(0,D\sigma_{w}^{2}\right);\varepsilon ∼N\left(0,I\sigma_{\varepsilon }^{2}\right);G=XX^{’}/m;D=ZZ^{’}/m$$ (2)

where **R** consisted of genomic inbreeding values (linear and quadratic) and τ were fixed effects associated to **R**. Significance of τ estimates was assessed by Wald tests. Model (2) was fitted in Ames-H by REML using the R package regress v1.3-15 (Clifford and McCullagh 2005).

#### Oligogenic effects (genome-wide association studies):

To assess marginal additive effects ($\beta_{j}$, fixed, for each SNP *j*), the following genome-wide association study (GWAS) model was fitted in Ames-H or NAM-H: $g=Q\delta +x_{j}\beta_{j}+u+\varepsilon$; $u∼N\left(0,G\sigma_{u}^{2}\right)$, $\varepsilon ∼N\left(0,I\sigma_{\varepsilon }^{2}\right)$, $G=XX^{’}/m$. Significance of SNPs was assessed by Wald tests on estimates of $\beta_{j}$. False discovery rates (FDRs) were estimated based on *P*-values from Wald tests by the method of Benjamini and Hochberg (1995). In addition, posterior inclusion probabilities (PIPs) were estimated by Bayesian sparse linear mixed models (BSLMMs), fitted jointly on the whole matrix **X** by GEMMA v0.98.1, with 1,000,000 and 10,000,000 MCMC iterations for burn-in and sampling, respectively (Zhou *et al.* 2013). Window posterior inclusion probabilities (WPIPs) were subsequently estimated, following Guan and Stephens (2011), by summing PIPs in 500-kb windows, sliding by 250-kb steps. The most significant marker effects were then selected for FDR ≤ 0.05 and WPIP ≥ 0.5.

To assess additive and dominance effects ($\beta_{j}$ and $\theta_{j}$, fixed, for each SNP *j*), GWAS models were extended in Ames-H to incorporate dominance for both fixed effects and random effects: $g=Q\delta +x_{j}\beta_{j}+z_{j}\theta_{j}+u+w+\varepsilon$; $u∼N\left(0,G\sigma_{u}^{2}\right)$, $w∼N\left(0,D\sigma_{w}^{2}\right)$, $\varepsilon ∼N\left(0,I\sigma_{\varepsilon }^{2}\right)$, $G=XX^{’}/m$, $D=ZZ^{’}/m$. Significance of SNPs was assessed by Wald tests on estimates of $\beta_{j}$ and $\theta_{j}$.

GWAS models were fitted under the efficient mixed-model association expedited (EMMAX) approximation of Kang *et al.* (2010), using function fastLm in the R package RcppEigen v0.3.3.5.0 (Bates and Eddelbuettel 2013).

#### Functional enrichments:

Effects of functional features on the amplitude of marker effects were captured by linear mixed models which partitioned the genomic variance by annotation bins. For each feature (gene proximity, recombination rate, chromatin openness, MAF, and GERP), the following functional enrichment model was fitted:

$$g=Q\delta +u+w+\varepsilon ;u∼N\left(0,\sum_{k}G_{k}\sigma_{k}^{2}\right);w∼N\left(0,\sum_{l}D_{l}\sigma_{l}^{2}\right);\varepsilon ∼N\left(0,I\sigma_{\varepsilon }^{2}\right);G_{k}=\frac{X_{k}X_{k}^{’}}{m_{k}},\mathrm{and}\;D_{l}=\frac{Z_{l}Z_{l}^{’}}{m_{l}}$$ (3)

where $X_{k}\left(Z_{l}\right)$ was the matrix of minor allele counts (heterozygosity) at the *m_k_* (*m_l_*) SNPs in bin *k* (*l*) and $\sigma_{k}^{2}\;\left(\sigma_{l}^{2}\right)$ was the variance component associated to additive effects in bin *k* (dominance effects in bin *l*). The significance of variance partitions was assessed by likelihood ratio tests, comparing the REML of the evaluated model to that of a baseline model. Model (3) was fitted by REML using the R package regress v1.3-15 (Clifford and McCullagh 2005) in Ames-H or NAM-H. In NAM-H, model (3) did not include dominance effects (**w**), because of collinearity of additive and dominance effects in this panel (due to only one inbred tester in NAM-H). Two types of variance partition were analyzed: partition by one feature (baseline: DGBLUP in Ames-H and GBLUP in NAM-H), and partition by both gene proximity and another feature (baseline: partition by gene proximity only in DGBLUP or GBLUP).

### Variance partition and SNP enrichment

The proportion of variance explained by marker effects in GBLUP was estimated by $\frac{1}{n}\sum_{i}\frac{{\tilde{G}}_{ii}\sigma_{u}^{2}}{{\tilde{G}}_{ii}\sigma_{u}^{2}+\sigma_{\varepsilon }^{2}}$, where ${\tilde{G}}_{ii}$ is the *i*^th^ diagonal element of matrix **G** adjusted for fixed effects, *i.e.*, $\tilde{G}=(I-H)G(I-H)$, with $H=Q{\left(Q^{\prime }Q\right)}^{-1}Q^{\prime }$ being the matrix of projection onto the column space of **Q**. The proportion of variance explained by additive marker effects in DGBLUP was estimated by $\frac{1}{n}\sum_{i}\frac{{\tilde{G}}_{ii}\sigma_{u}^{2}}{{\tilde{G}}_{ii}\sigma_{u}^{2}+{\tilde{D}}_{ii}\sigma_{w}^{2}+\sigma_{\varepsilon }^{2}}$, and similarly for dominance effects, $\frac{1}{n}\sum_{i}\frac{{\tilde{D}}_{ii}\sigma_{w}^{2}}{{\tilde{G}}_{ii}\sigma_{u}^{2}+{\tilde{D}}_{ii}\sigma_{w}^{2}+\sigma_{\varepsilon }^{2}}$ [model (1)], with $\tilde{D}=(I-H)D(I-H)$. In functional enrichment models, SNP enrichment for additive effects at bin k* was calculated by the ratio of $\left[\frac{1}{n}\underset{i}{\sum }{\tilde{G}}_{k*ii}\sigma_{k^{*}}^{2}\right]/\left[\frac{1}{n}\underset{i}{\sum }\left(\underset{k}{\sum }{\tilde{G}}_{kii}\sigma_{k}^{2}+\underset{l}{\sum }{\tilde{D}}_{lii}\sigma_{l}^{2}\right)\right]$, *i.e.*, the proportion of genomic variance explained by bin k*, over $m_{k^{*}}/\left[\underset{k}{\sum }m_{k}+\underset{l}{\sum }m_{l}\right]$, *i.e.*, the proportion of SNP effects in bin k* (and similarly for dominance effects at bin l*) [model (3)].

### Validation of genomic prediction models

Prediction accuracy of genomic models (GBLUP, DGBLUP, GWAS models, and functional enrichment models) was characterized by the Pearson correlation between observed genotype means and their predicted values in validation sets. For models trained in Ames-H, a validation set was 1 of the 24 NAM-H populations. This validation scheme assessed prediction models for genomic selection in biparental breeding populations, and was compared to a “leave-one-population-out” cross-validation scheme where, for each validation set (population), models were trained on the remaining 23 populations in NAM-H. For models trained in NAM-H, a validation set was 1 of 10 random subsets in Ames-H. These subsets were created by a random partition stratified by tester and population cluster (Supplemental Material, Figure S1), so that all validation sets equally represented variation over population clusters and testers. This validation scheme assessed prediction models for evaluation in diverse panels. The significance of prediction accuracy for any genomic model was tested for nonzero mean (by a one-sample Student’s *t*-test) and difference to another model (by a two-sample Student’s *t*-test paired by validation set).

### Assessment of genotype-by-panel interactions

Interactions between genotypes and panels were assessed by Pearson correlation in genotype means between panels for hybrids common to both panels $\left(\rho_{R}\right)$. These hybrids were derived from crosses between PHZ51 and 1 of 23 reference lines (B73, B97, CML52, CML69, CML103, CML228, CML247, CML277, CML322, CML333, Il14H, Ki3, Ki11, M162W, M37W, Mo17, Mo18W, NC350, NC358, Oh43, P39, Tx303, and Tzi8).

Genotype-by-panel interactions were also assessed by the following polygenic model, following Jarquín *et al.* (2014):

$$g=\tilde{Q}\tilde{\delta }+\tilde{u}+\varepsilon ,\tilde{u}∼N\left(0,G\sigma_{0}^{2}+\left[G○EE^{'}\right]\sigma_{1}^{2}\right),\varepsilon ∼N\left(0,I\sigma_{\varepsilon }^{2}\right),G=XX^{’}/m$$

where **E** was the *n* × 2 design matrix attributing genotypes to panels, either Ames-H or NAM-H; $\tilde{Q}=\left[EP\right]$ and $\tilde{\delta }$ captured effects of panels and population structure; $\tilde{u}$ were polygenic genomic effects with main variance and panel-specific variance being quantified by $\sigma_{0}^{2}$ and $\sigma_{1}^{2}$, respectively; and ○ refers to the Hadamard (element-wise) product. For a given hybrid *i*, correlation in ${\tilde{u}}_{i}$ between different panel *j* and *j*’ was defined by $\rho_{G}=\text{Cor}\left({\tilde{u}}_{ij},{\tilde{u}}_{ij^{’}}\right)=\frac{G_{ii}\sigma_{0}^{2}}{G_{ii}\left(\sigma_{0}^{2}+\sigma_{1}^{2}\right)}=\frac{\sigma_{0}^{2}}{\sigma_{0}^{2}+\sigma_{1}^{2}}$ (Jarquín *et al.* 2014). This model was fitted in Ames-H and NAM-H jointly, by REML using the R package regress v1.3-15 (Clifford and McCullagh 2005).

### Data availability

Supporting data can be downloaded from https://doi.org/10.25739/x2wc-yj71: raw marker scores at GBS SNPs (Ames and NAM inbred panels), imputed marker scores at WGS SNPs (Hapmap 3.2.1, Ames-H, and NAM-H), raw phenotypic measurements (Ames-H and NAM-H), estimated genotype means (Ames-H and NAM-H), and functional annotations. Raw marker scores at WGS SNPs in Hapmap 3.2.1 are publicly available (https://doi.org/10.7946/p28h0c; Bukowski *et al.* 2018). Supporting code (in R and Bash) is available at https://bitbucket.org/bucklerlab/ames_nam_hybrid. Supplemental material available at figshare: https://doi.org/10.25386/genetics.11952225. Table S1 describes interactions between genotypes and panels. Table S2 describes high-confidence QTL effects in Ames-H and NAM-H, based on GWAS models and BSLMMs. Table S3 shows summary statistics about genomic inbreeding in Ames-H. Table S4 describes the difference in accuracy between Ames-H subsets (Ames × PHZ51 and Ames × B47) for prediction in NAM-H. Table S5 describes the prediction accuracy in NAM-H by directional effects of inbreeding in Ames-H. Table S6 describes the prediction accuracy of GWAS models. Table S7 presents the significance of variance partition by panel and functional feature. Table S8 describes the genomic heritability captured by functional classes in functional enrichment models. Table S9 describes the genomic prediction accuracy of functional enrichment models. Figure S1 shows the population clusters in Ames-H, inferred by *k*-means clustering (*k* = 4). Figure S2 depicts linkage disequilibrium (LD) in Ames-H and NAM-H. Figure S3 shows allele frequencies among female parents in Ames-H and NAM-H. Figure S4 shows the significance of marginal additive effects in GWAS in Ames-H and NAM-H. Figure S5 shows the SNP enrichment by bin for structural and evolutionary features in Ames-H and NAM-H. Figure S6 shows the SNP enrichment by bin for structural and evolutionary features in Ames-H and NAM-H, while accounting for enrichment by gene proximity.

## Results

### Hybrid panels differ by genetic diversity and genetic effects for GY

#### Hybrid panels display contrasting levels of diversity:

**Figure 2:**
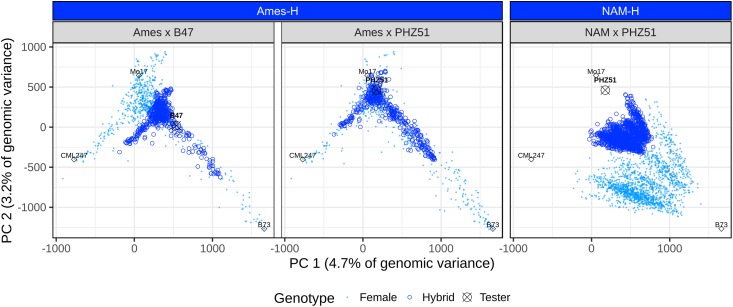
The two hybrid panels differ by their number of testers and their level of diversity. PC plot of hybrids. Ames-H consists of crosses between the Ames inbred panel and B47 (Ames × B47) or PHZ51 (Ames × PHZ51). NAM-H consists of crosses between the NAM inbred panel and PHZ51. Crossed circles refer to the male tester inbred line; light blue dots refer to female inbred lines; and dark blue open circles refer to the hybrid lines making up Ames-H and NAM-H. Diamonds refer to reference lines: B73, SS reference line; Mo17, NSS reference line; and CML247, CIMMYT semitropical reference line. PCs and proportions of genomic variance explained were computed based on WGS SNPs in the Goodman panel. Ames, North Central Regional Plant Introduction Station association panel; Ames-H, hybrid Ames panel; NAM, U.S. Nested Association Mapping panel; NAM-H, hybrid NAM panel; PC, principal component; WGS, whole-genome sequencing.

While the Ames hybrid panel (Ames-H) has a few hybrids with an affinity to semitropical lines like CML 247, it is, for the most part, comprised of hybrids closely related to SS lines like B73 and NSS lines like Mo17 (Figure 2). Compared to Ames-H, NAM-H is less diverse, since it was produced by crosses between a single NSS tester (PHZ51) and biparental populations that were all derived from a cross involving B73 as a common parent (NAM RILs are 50% B73). Moreover, female parents in NAM-H were selected for similar flowering time to PHZ51, hence further narrowing down the genetic diversity in this panel.

#### Genome-wide patterns of LD and allele frequency are similar across panels:

LD patterns are quite similar in both hybrid panels. After adjustment for population structure and relatedness (following Mangin *et al.* 2012), LD values are moderately concordant between Ames-H and NAM-H (*r =* 0.77) (Figure S2). Average LD values along chromosomes decay at similar rates, reaching 0.1 at 160 kb in Ames-H and 151 kb in NAM-H. Allele frequencies among female parents are also concordant between Ames-H and NAM-H (*r* = 0.88), but there are SNPs at low frequency in NAM-H (< 0.5) that segregate at frequencies between 0 and 1 in Ames-H (Figure S3).

#### Genetic architecture for GY differs across panels:

Three agronomic traits were analyzed in Ames-H and NAM-H: DTS, PH, and GY adjusted for differences in flowering time among hybrids. The relatively low accuracy of genotype means for GY (as reflected by low broad-sense heritability and entry-mean reliability; Table 1) suggests variability due to genotype-by-environment interactions. Accordingly, genotypic effects for GY appear highly inconsistent across panels (Table S1). For GY, correlations across panels based on genotype means of reference lines $\left(\rho_{R}\right)$ and genomic marker effects $\left(\rho_{G}\right)$ are not significantly different from zero (*P*-value > 0.10; Table S1). In contrast, consistency in genetic effects is higher for PH ($\rho_{R}=0.65$ and $\rho_{G}=0.78$; *P*-value < 0.001) and DTS ($\rho_{R}=0.93$ and $\rho_{G}=1.0$; *P*-value < 0.001) (Table S1). Therefore, DTS, PH, and GY represent three distinct levels of sensitivity to genotype-by-environment interactions, being respectively weak, moderate, and strong.

### Evidence for dominance effects in hybrids is consistent for PH and GY

Analyses of dominance were conducted in Ames-H only. Under the hypothesis that dominance effects are pervasive in Ames-H, we expected consistent evidence for different types of dominance effects: polygenic effects from variance partition [*Materials and Methods*: model (1)], fixed QTL effects from association mapping, and directional effects from analysis of genomic inbreeding [*Materials and Methods*: model (2)]. Furthermore, we expected gains in genomic prediction accuracy to be achieved by any type of dominance effects in an extraneous panel (NAM-H) (Figure 1). Dominance effects of markers were not estimated in NAM-H because hybrids in this panel are characterized by only two genotype classes at any given marker (instead of three in Ames-H), such that additive and dominance effects are statistically equivalent in NAM-H.

#### Polygenic dominance effects capture genotypic variability for all traits:

**Figure 3:**
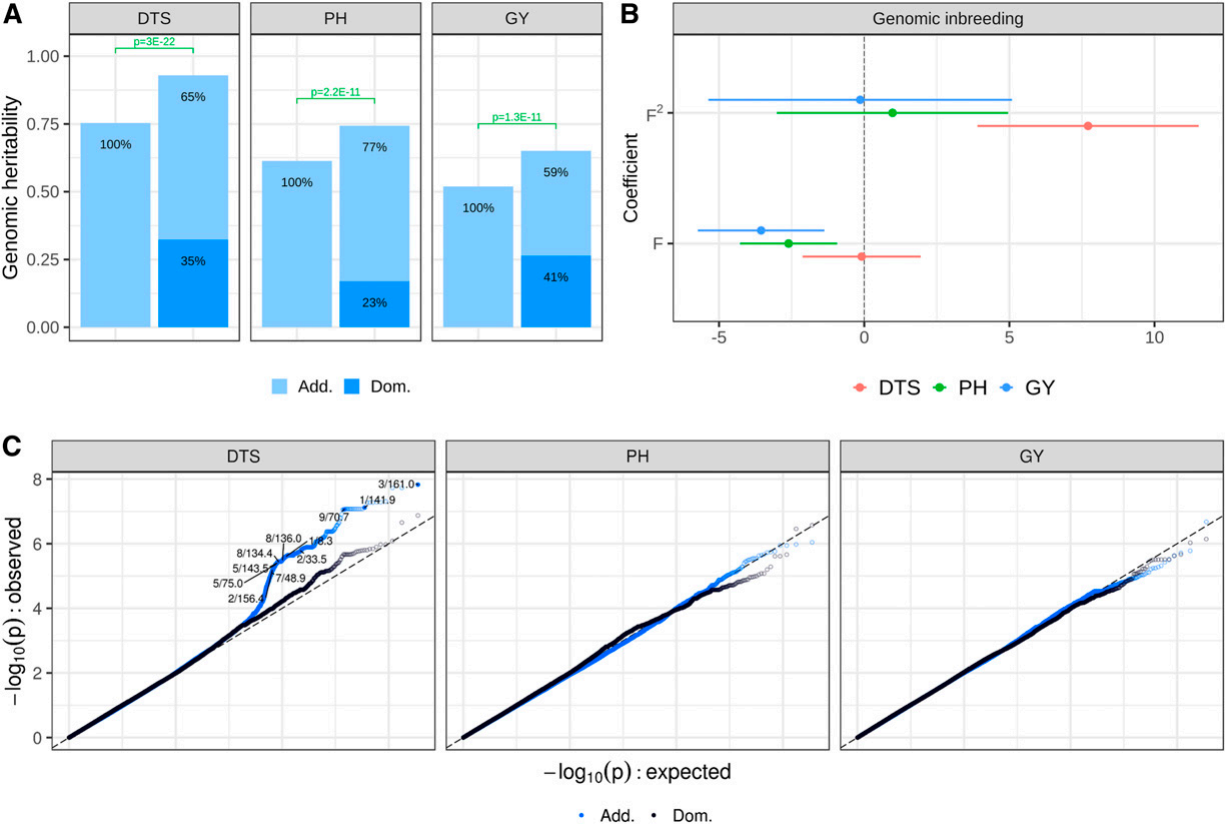
Dominance genetic effects are prevalent for PH and GY, but not for DTS, in Ames-H. (A) Partition of variance by additive (Add.) and dominance (Dom.) effects in genome-wide polygenic models; genomic heritability: proportion of variance among genotype means captured by Add. or Dom. marker effects; and p: *P*-values from likelihood ratio tests. (B) Estimated effects of genomic inbreeding (point and 95% C.I.). Effects are shown in unit of SD for each trait. F: linear effect; F^2^: quadratic effect. (C) Quantile–quantile plot for joint estimates of Add. and Dom. effects in GWAS. Effects of SNPs were deemed significant if their FDR was ≤ 0.05 and if they were not within 1 Mb of SNPs with more significant effects (effects with lower *P*-values). SNPs with significant effects are designated by chromosome number and genomic position in megabases. Ames-H, hybrid North Central Regional Plant Introduction Station association panel; FDR, false discovery rate; DTS, days to silking; PH, plant height; GY, grain yield adjusted for DTS.

To assess the statistical relevance of polygenic dominance effects, genotypic variability was partitioned into additive and dominance components in a DGBLUP model. For all traits, dominance accounts for a significant portion of genotypic variability in Ames-H (*P*-value ≤ 2.2 × 10^−11^), capturing 35, 23, and 41% of genomic variance for DTS, PH, and GY, respectively (Figure 3A). These estimates correspond to average degrees of dominance (ratio of dominance-to-additive SD) of 0.73, 0.54, and 0.83 respectively. Therefore, overdominance does not seem to be pervasive in Ames-H (average degrees of dominance < 1).

#### Effects of QTL are significant for DTS, but do not suggest dominance gene action:

GWAS models reveal multiple significant QTL effects for DTS (Figure S4). There are five and seven high-confidence QTL (FDR ≤ 0.05 and WPIP ≥ 0.5; see *Materials and Methods*) for DTS in Ames-H and NAM-H, respectively (Figure S4 and Table S2). For PH and GY, no QTL effects are significant except for one QTL in NAM-H for GY (Table S2). GWAS signals for DTS show limited consistency between Ames-H and NAM-H, with no overlap of high-confidence QTL across panels (Figure S4 and Table S2).

To test whether dominance contributes to QTL effects, we conducted a GWAS for additive and dominance QTL effects in Ames-H. Multiple additive effects appear significant for DTS, with significant QTL effects (FDR ≤ 0.05) in chromosomes 3, 1, and 9 (Figure 3C). But, dominance effects are not significant (FDR > 0.30) (Figure 3C), so the inconsistency in QTL effects for DTS across panels probably does not involve dominance. Moreover, genetic effects for DTS do not appear to be sensitive to environments (Table S1), and there are no systematic differences in allele frequency that consistently explain differences in significance of QTL effects across panels (Table S2). Thus, it is plausible that higher-order genetic interactions (epistasis) cause the difference in QTL significance for DTS across panels.

#### Effects of inbreeding point to higher-order genetic interactions for DTS:

Under directional dominance, inbreeding should be linearly related to fitness, but such relationship will tend to be nonlinear under higher-order epistatic interactions such as dominance × dominance interactions (Crow and Kimura 1970). To test whether dominance contributes to genotypic variability by directional effects, we assessed linear and quadratic effects of genomic inbreeding (*F*) on agronomic traits. For PH and GY in Ames-H, only linear effects of genomic inbreeding are significant (Figure 3B and Table S3). Moreover, these effects are on par with their expected impact on fitness, since genomic inbreeding is negatively associated with PH and GY. For DTS in Ames-H, only the quadratic effect of genomic inbreeding is significant (Figure 3B). Along with the lack of dominance QTL effects, this lack of linear effects suggests that nonadditive genetic effects for DTS may not be properly captured by dominance effects, contrary to PH and GY where evidence from polygenic and directional effects is consistent.

#### Polygenic dominance effects increase prediction accuracy for PH:

Prediction accuracy within NAM-H (in leave-one-population-out cross-validation) is consistently higher than prediction accuracy from Ames-H to NAM-H, especially for GY (Table 2). Indeed, the observed decreases in prediction accuracy (between GBLUP trained in Ames-H and GBLUP trained in NAM-H) are larger when genomic correlations across panels are lower (Table S1). For DTS and PH, GBLUP models trained in Ames-H (1106 hybrid crosses with PHZ51 or B47) are significantly more accurate than those trained in Ames × B47 (subset of 643 hybrid crosses with B47 only), highlighting the benefit of having a similar tester (PHZ51) between training and validation panels. Furthermore, GBLUP models trained in Ames-H are as or more accurate than those trained in Ames × PHZ51 (subset of 463 hybrid crosses with PHZ51 only), which demonstrates the robustness of GBLUP models to multiple testers within training sets. For GY, a GBLUP model trained in Ames × B47 is more accurate than those trained in Ames-H or Ames × PHZ51 (Table 2). The close genetic relationship between B47 and the common parent in NAM-H, B73, may benefit prediction accuracy from Ames × B47 to NAM-H, despite the difference in tester (Figure 2). Moreover, the gain in prediction accuracy from Ames × PHZ51 to Ames × B47 is only significant in NAM-H populations that originate from tropical lines, at equal sample sizes (+0.124; *P*-value = 0.010; Table S4); therefore, female parents in Ames × B47 may also contribute to better predictions for GY, especially in NAM-H populations of tropical origin.

**Table 2:** Genomic prediction accuracy in NAM-H

	Cross-validation prediction accuracy (*P*-value)	Prediction accuracy (*P*-value)	Difference in prediction accuracy (*P*-value)		
Training panel (Testers)	NAM-H (PHZ51)	Ames-H (PHZ51, B47)	Ames-H (PHZ51, B47)	Ames × PHZ51 (PHZ51 only)	Ames × B47 (B47 only)
Model	GBLUP	GBLUP	DGBLUP	GBLUP	GBLUP
DTS	0.405 (2.2 × 10^−11^)	0.331 (2.3 × 10^−11^)	−0.013 (0.21)	−0.028 (0.078)	−0.068 (2.7 × 10^−3^)
PH	0.394 (1.4 × 10^−10^)	0.235 (9.6 × 10^−8^)	+0.023 (0.021)	+0.014 (0.42)	−0.095 (8.5 × 10^−5^)
GY	0.240 (2.2 × 10^−9^)	−0.001 (0.96)	−0.010 (0.33)	−0.018 (0.29)	+0.056 (0.014)

Prediction accuracy: average correlation between observed and predicted phenotypes over the 24 populations in NAM-H; cross-validation prediction accuracy: leave-one-population-out prediction accuracy within NAM-H; difference in prediction accuracy: difference in cross-panel prediction accuracy between models (DGBLUP *vs.* GBLUP model in Ames-H) or between training sets (Ames × PHZ51 or Ames × B47 *vs.* Ames-H). Significance of average prediction accuracies (nonzero mean) and estimated differences in prediction accuracy (nonzero difference, paired by NAM-H population) was assessed by Student’s *t*-tests. Ames, North Central Regional Plant Introduction Station association panel; Ames-H, hybrid Ames panel; NAM, U.S. Nested Association Mapping panel; NAM-H, hybrid NAM panel; GBLUP, genomic best linear unbiased prediction; DGBLUP, dominance GBLUP; DTS, days to silking; PH, plant height; GY, grain yield adjusted for DTS.

Incorporating dominance effects in GBLUP models results in significant gains in prediction accuracy for PH only (+0.023; *P*-value *=* 0.021), and no significant differences for DTS and GY (Table 2). Therefore, accounting for polygenic dominance effects should not be detrimental to genomic prediction models and may even increase their accuracy. In contrast, fixed effects do not contribute to increased prediction accuracy: directional effects from genomic inbreeding result in small and nonsignificant differences in prediction accuracy (Table S5); additive and dominance effects at high-confidence QTL in GWAS models result in large and moderately significant decreases in prediction accuracy for DTS (−0.059; *P*-value < 0.10; Table S6).

### Heterogeneity in polygenic effects is best captured by gene proximity

Analyses of functional features (gene proximity, recombination rate, chromatin openness, MAF, and GERP scores) were conducted in Ames-H and NAM-H. Under the hypothesis that there are differential effects across functional classes, we expected SNP enrichment (enrichment in genomic heritability by class of SNPs) in functional enrichment models (partition of additive and dominance variance in Ames-H, and partition of additive variance in NAM-H) [*Materials and Methods*: model (3)]. Moreover, we expected gains in prediction accuracy achieved by functional enrichment models across panels (Figure 1).

#### Polygenic effects are enriched in genic regions for all traits:

**Figure 4:**
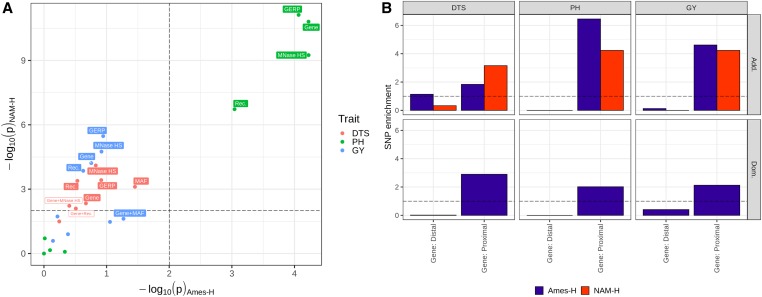
Effects of SNPs are enriched in gene-proximal regions for DTS, PH, and GY, in Ames-H and NAM-H. (A) Significance of variance partition by gene proximity (Gene), structural features (Rec. and MNase HS) or evolutionary features (MAF and GERP), and variance partition after accounting for gene proximity (Gene+Rec., Gene+MNase HS, Gene+MAF, and Gene+GERP); *P*-values (p) were obtained by likelihood ratio test comparing the functional enrichment model to a baseline model with no partition for the feature of interest (*e.g.*, Gene *vs.* unpartitioned model or Gene+MAF *vs.* Gene); dashed lines correspond to thresholds for significance in either panel, after adjustment by Bonferroni correction. Labels refer to significant features after Bonferroni correction, based on *P*-values in either panel (open boxes) or *P*-values in both panels combined by Fisher’s method (full boxes) (Table S7). (B) SNP enrichment (inflation of SNP effects by functional class, *i.e.*, the ratio of the proportion of genomic variance explained over the proportion of SNP effects), for additive (Add.) and dominance (Dom.) effects, by bin for gene proximity (Gene). Proximal: ≤1 kb of an annotated gene; Distal: > 1 kb away from an annotated gene. Ames-H, hybrid North Central Regional Plant Introduction Station association panel; NAM-H, hybrid U.S. Nested Association Mapping panel; GERP, genomic evolutionary rate profiling; MAF, minor allele frequency; MNase HS, micrococcal nuclease hypersensitivity; Rec., recombination rate; DTS, days to silking; PH, plant height; GY, grain yield adjusted for DTS.

Partition of genomic variance by proximity to annotated genes is significant for all traits in Ames-H and NAM-H, based on likelihood ratio tests combined by Fisher’s method (*P*-value < 0.01; Figure 4A and Table S7). As suggested by the high correlation in significance [−log_10_(*P*-value)] between Ames-H and NAM-H (*r =* 0.92), the higher significance of partitions in NAM-H could be due to a systematic increase in statistical power, due in part to the larger sample size in NAM-H (*n* = 1640 *vs.* *n* = 1106).

Polygenic SNP effects are consistently enriched near genic regions (Figure 4B), and the proportion of variance explained by gene-proximal SNPs is consistently larger than explained by gene-distal SNPs (56–100% of the genomic variability is explained by gene-proximal SNPs, across traits and panels; Table S8). Therefore, genetic effects seem enriched in genic regions, especially for PH where enrichment is highly significant in both panels.

#### Enrichment of polygenic effects by structural and evolutionary features is unclear:

Partition of genomic variance explained by recombination rate, chromatin openness, MAF, and GERP scores is significant, for DTS in NAM-H only, for PH in both panels (except MAF), and for GY in NAM-H only (except MAF) (Figure 4A and Table S7). SNP enrichments in both panels indicate that the magnitudes of polygenic effects tend to be larger at low-diversity loci (low MAF and high GERP scores) and in euchromatic regions (open chromatin and moderate-to-high recombination rates) (Figure S5). However, none are significant across traits after accounting for gene proximity, based on likelihood ratio tests combined by Fisher’s method (*P*-value > 0.01; Table S7). Because evolutionary constraint and chromatin openness are positively associated with gene density, enrichment at these features may be due to functional enrichment by gene proximity (Figure S6). One notable exception is MAF for GY (*P*-value = 9.8 × 10^−3^; Table S7), which indicates some promise for SNP enrichment by MAF classes. However, enrichments by MAF classes are inconsistent across panels, with rare SNPs (MAF ≤ 0.01) being functionally enriched only in NAM-H, for example (Figure S6).

#### Variance partition by gene proximity or GERP scores increases prediction accuracy for all traits:

**Figure 5:**
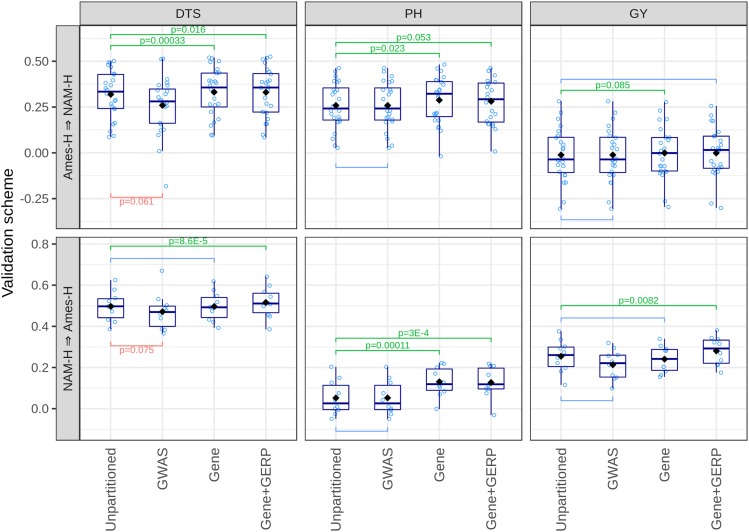
Functional enrichment by gene proximity and GERP scores improves accuracy of genomic prediction models for DTS, PH, and GY. Prediction accuracy (*y*-axis): average correlation between observed and predicted genotype means in validation sets. Validation scheme: training panel ⇒ validation panel. Unpartitioned: DGBLUP (polygenic additive and dominance effects) in Ames-H, GBLUP (polygenic additive effects only) in NAM-H; GWAS, fixed effects at high-confidence QTL from GWAS; Gene, functional enrichment by proximity to genes (≤1 kb of an annotated gene); Gene+GERP, functional enrichments by proximity to genes and GERP scores. Black diamonds indicate average prediction accuracy. Significance of estimated differences in prediction accuracy (nonzero difference) was assessed by Student’s *t*-tests, paired by validation set. Only *P*-values < 0.1 (p) are shown. Ames-H, hybrid North Central Regional Plant Introduction Station association panel; NAM-H, hybrid U.S. Nested Association Mapping panel; GERP, genomic evolutionary rate profiling; GBLUP, genomic best linear unbiased prediction; DGBLUP, dominance GBLUP; DTS, days to silking; PH, plant height; GY, grain yield adjusted for DTS.

Partitioning genomic variance by gene proximity is often useful to genomic prediction. From Ames-H to NAM-H (training in Ames-H and validation in NAM-H), partition of genomic variance by gene proximity increases prediction accuracy for DTS (+0.013, *P*-value *=* 3.3 × 10^−4^), PH (+0.029, *P*-value = 0.023), and GY (+0.010, *P*-value = 0.085), compared to a DGBLUP model (Figure 5). From NAM-H to Ames-H, partition by gene proximity increases prediction accuracy for PH only (+0.078, *P*-value = 1.1 × 10^−4^), compared to a GBLUP model (Figure 5). Other features than gene proximity result in further gains in prediction accuracy (Table S9). In particular, partitioning genomic variance by GERP scores is useful for genomic prediction, even when accounting for enrichment by gene proximity, with significant gains in prediction accuracy achieved from NAM-H to Ames-H for DTS (+0.019, *P*-value *=* 1.6 × 10^−4^) and GY (+0.040, *P*-value = 5.4 × 10^−6^) (Figure 5 and Table S9). There are also some improvements by taking into account recombination rate (+0.014 for GY from Ames-H to NAM-H, and +0.013 for DTS and +0.020 for GY from NAM-H to Ames-H), but they are less substantial than those achieved by using GERP scores (Table S9). Even when classes based on MNase HS and GERP scores do not yield significant gains in prediction accuracy, the high SNP enrichments achieved by taking into account these features (∼32-fold for open-chromatin regions and ∼8-fold for GERP scores > 0) can be of practical interest for SNP prioritization in genomic prediction (Figure S5 and Table S8).

Improvements of prediction accuracy by enrichment of SNP effects in functional classes contrast with the lack of improvement from enrichment of SNP effects by GWAS. Incorporating high-confidence QTL effects from GWAS as fixed effects in either a DGBLUP model (from Ames-H to NAM-H) or a GBLUP model (from NAM-H to Ames-H) does not improve prediction accuracy, regardless of traits or validation schemes (Figure 5 and Table S6). These results suggest that prioritizing few statistically significant loci (based on GWAS) may not be as useful as prioritizing broader classes of loci, probably because of background dependency of strong QTL effects (marker-by-population and marker-by-environment interactions).

## Discussion

### Do additive and dominance effects adequately capture genetic architectures?

For all traits, a significant proportion of variance is explained by dominance effects (Figure 3A). However, for DTS, there is conflicting evidence about the importance of dominance: (i) no significant dominance QTL effects despite significant additive QTL effects (Figure 3C) and (ii) significant quadratic effects of genomic inbreeding, without any linear effect (Figure 3B). Such evidence indicates that DTS should probably be analyzed under more complex genetic models involving epistatic interactions, possibly reflecting the complex molecular pathways underlying flowering time (*e.g.*, photoperiod genes; Yang *et al.* 2013; Blümel *et al.* 2015; Minow *et al.* 2018). In this study, genomic variance in Ames-H could not be partitioned reliably by additive, dominance, and epistatic effects, because genomic relationships for pairwise epistatic effects are highly correlated with those for additive effects (*r* > 0.99 between additive and additive × additive relationships). Moreover, epistatic effects in linear mixed models vary depending on how marker variables are centered, in a way that can be arbitrary (Martini *et al.* 2016, 2017). However, further analyses to investigate the contribution of epistatic effects to genomic variance is merited (Jiang and Reif 2015). Investigating epistatic effects would likely require large panels with more testers, and also efficient methodologies to restrict the number of interactions (*e.g.*, only interactions between homeologs; Santantonio *et al.* 2019) and the types of effects involved (*e.g.*, only SNP × SNP interactions like additive × additive effects, or SNP × background interactions like SNP × PC effects; Ramstein *et al.* 2018).

For PH and GY, there is consistent evidence for prevalent dominance effects: (i) significant variance partition by dominance effects (Figure 3A) and (ii) significant linear effects of genomic inbreeding, without any quadratic effect (Figure 3B). Therefore, additive and dominance effects may efficiently capture genetic effects for PH and GY. These results contrast with previous studies on hybrid maize, which showed that additive effects could capture most of genotypic variability. Critically, those studies were based on panels derived solely from crosses between different heterotic groups (due to the practical relevance of such crosses), *e.g.*, Flint × Dent (Technow *et al.* 2014; Giraud *et al.* 2017) or SS × NSS (Kadam *et al.* 2016). Therefore, complementation effects were relatively consistent across hybrids and well captured by general combining abilities, such that variability for specific combining ability was low. In contrast, one of our panels (Ames-H) shows strong variation for complementation effects because it represents a variety of genetic contexts (SS × NSS, SS × SS, Semitropical × SS, etc.). Therefore, it is better suited to represent the differential levels of complementation effects in maize and reveal the importance of dominance effects across maize hybrids.

No QTL effects were detected in Ames-H for PH or GY, whereas previously published analyses have reported significant additive and dominance QTL effects for these traits (Schön *et al.* 2010; Larièpe *et al.* 2012). Those studies were based on populations from Design III experiments, where biparental progeny (typically RILs) are crossed with either parent. In comparison, panels like Ames-H or even NAM-H are genetically more diverse, and are characterized by many low-frequency variants (Figure S3). So, heterogeneity in genetic backgrounds and low variability at markers likely contribute to lower QTL detection power in Ames-H and NAM-H. Moreover, it is possible that the larger QTL effects detected in Design III studies arose from multiple loci in strong linkage, due to relatively few (effective) recombination events in their populations. Consistently, degrees of dominance estimated in those studies were especially large around centromeres, possibly because of repulsion-phase linkage (Hill and Robertson 1966). In contrast, genome-wide degrees of dominance estimated in Ames-H are relatively low (< 1) and no significant enrichment for dominance effects is detected in low-recombination regions, after accounting for gene proximity (Table S7). Therefore, differences in QTL detection power between Ames-H and Design III populations may be caused by important differences in both genetic diversity and LD structure.

### What is the biological basis for enrichment of SNP effects by gene proximity?

Analyses of functional enrichment point to genetic effects arising mostly from genic regions (proximal SNPs, ≤ 1 kb from annotated genes). The relevance of genic regions for capturing genotypic variability in hybrid maize is consistent with hypotheses about biological causes of heterosis (hybrid vigor) related to gene expression, namely, (i) nonadditive inheritance of gene expression and (ii) nonlinear effects of gene expression on agronomic traits (Springer and Stupar 2007; Schnable and Springer 2013). Proposed mechanisms for nonadditive inheritance of gene expression include complementation with respect to regulatory motifs or transcription factors, and presence/absence variation (Paschold *et al.* 2012; Marcon *et al.* 2017; Zhou *et al.* 2019), but studies in maize generally report that most genes have an additive mode of inheritance for expression levels (*e.g.*, Stupar and Springer 2006; Swanson-Wagner *et al.* 2006; Zhou *et al.* 2019). Conversely, the gene balance hypothesis indicates that highly connected genes (in pathways, protein complexes, etc.) should be expressed in relative amounts under a stoichiometric optimum (Birchler and Veitia 2010). This optimum points to a balance between benefits of gene expression (from RNA or protein activity) and its costs (from energy requirements and accumulation of wasteful products, or toxic intermediates), which may result in nonlinear effects of gene expression (Dekel and Alon 2005; Domingo *et al.* 2019). Results in support of the gene balance hypothesis in maize include intermediate gene expression harboring minimal burden of deleterious mutations in diverse maize inbred lines (Kremling *et al.* 2018). Furthermore, models related to metabolic fluxes provide examples of mechanisms by which gene balance can arise (Fiévet *et al.* 2010; Vacher and Small 2019). These models describe the nonlinear relationships between enzyme concentrations and metabolic fluxes (*e.g.*, hyperbolic functions), so that additive genetic effects on gene expression may translate into nonadditive effects on agronomic traits. Importantly, benefits and costs of gene expression are affected by intragenic interactions (*e.g.*, protein folding; Otwinowski *et al.* 2018), which can be captured by dominance effects and LD within genes, but also intergenic interactions (*e.g.*, pathway interactions or protein–protein interactions; Diss and Lehner 2018; Vacher and Small 2019), which cannot be captured predictably by dominance effects. Ideally, future research about nonadditive genetic effects and their enrichment in genic regions will involve transcriptomic, proteomic, and/or metabolomic data, to empirically test the gene balance hypothesis and provide mechanistic explanations for genotypic variability in hybrid maize.

### Are dominance effects and enrichments in genic regions useful for genomic prediction?

In this study, the relevance of dominance effects and functional enrichments was evaluated by genomic prediction *across* panels. Therefore, prediction models were assessed for their ability to sustain accuracy across distinct population backgrounds. Genomic prediction accuracy was estimated in NAM-H to reflect genomic selection applications in biparental breeding populations crossed to a single tester. These populations serve as useful validation sets because they exemplify interheterotic populations, as crosses between PHZ51 (an NSS line) and NAM inbred lines (related to the SS pool by ≥ 50% through the common B73 parent). Therefore, these populations are good examples of breeding populations crossed to a relevant tester. In contrast, genomic prediction accuracy was estimated in Ames-H to reflect a different situation where diverse panels are evaluated for hybrid performance, in genomic prospection (*e.g.*, for prebreeding applications; Voss-Fels *et al.* 2019). Under this validation scheme, validation sets do not correspond to breeding populations, but instead consist of diverse hybrid panels structured by subpopulations and testers (Figure S1).

Enrichment of SNP effects emphasizes causal loci; therefore, enrichment procedures such as QTL detection or variance partition can improve the accuracy of genomic prediction models. However, as genetic effects vary between populations, enrichments about small functional classes (*e.g.*, a few GWAS hits) lose their potential. This caveat is exemplified by differences in QTL effects for DTS between Ames-H and NAM-H, and the consequent lack of gain in accuracy by prediction models based on QTL effects (Figure 5 and Table S6). Similarly, Spindel *et al.* (2016) showed benefits of major QTL effects for prediction of flowering time in rice, but only when QTL were detected in the target breeding populations. Contrary to enrichments for QTL, enrichments for larger functional classes (*e.g.*, gene-proximal SNPs) should result in gains of prediction accuracy that are robust to differences in population backgrounds, as is observed here (Figure 5 and Table S9). Likewise, Gao *et al.* (2017) reported gains in genomic prediction accuracy by prioritizing genic SNPs in mice, *Drosophila*, and rice (increases in predictive ability averaging +0.013, similar to those realized in this study). Therefore, gains in prediction accuracy by gene proximity should be expected in a broad range of population and species contexts.

While functional enrichments by gene proximity appeared beneficial for all traits, incorporating polygenic dominance effects resulted in gains in prediction accuracy for PH only (Table 2). The lack of gain in prediction accuracy for DTS and GY illustrates possible reasons for disagreement between quality of fit and prediction accuracy often observed in genomic prediction studies. For DTS, incorporating dominance effects results in *statistically* significant improvements in fit, but a genetic model accounting for epistatic interactions appears more plausible according to analyses of QTL and genomic inbreeding. Thus, the choice of prediction procedure should probably come from multiple pieces of evidence in favor of a given genetic model, rather than a single statistical test about the genomic prediction model. In the case of GY, prediction accuracy across panels is probably hindered by genotype-by-environment interactions, which could be accommodated by models incorporating environmental covariates (*e.g.*, Li *et al.* 2018; Millet *et al.* 2019).

### Conclusions

Our analyses point to genetic models in hybrid maize that involve interactive effects and emphasize genic regions. While dominance may be relevant to all three traits, other nonlinear effects seemed important for DTS and interactions with environments appeared critical for GY. Consistently, genomic prediction models were improved by dominance effects for PH only. In contrast, genomic prediction models benefited from functional enrichment in genic regions for all traits. Although gene proximity appeared most useful and meaningful in our study, the value of structural and evolutionary features for genomic prediction deserves more attention. Our results call for further investigation about the biological basis of genetic complementation and the underlying interactive effects that could enable more robust prediction of genotypic variability in hybrid maize.
